# Supplementary material for: Structural modeling of the hERG potassium channel and associated drug interactions
Source: Front Pharmacol. 2022 Sep 16;13:966463. doi: 10.3389/fphar.2022.966463 (PMC9523588; doi:10.3389/fphar.2022.966463)
Supplement: Supplementary file 1 [file DataSheet1.PDF]

Supplemental Material for:

Structural modeling of the hERG potassium channel and associated drug interactions

Jan Maly, Aiyana M. Emigh, Kevin R. DeMarco, Kazuharu Furutani, Jon T. Sack, Colleen E. Clancy, Igor Vorobyov[1], Vladimir Yarov-Yarovoy[1]

Department of Physiology and Membrane Biology (J.M., A.M.E, K.R.D, K.F., J.T.S, C.E.C, I.V, V.Y.-Y.), Department of Anesthesiology and Pain Medicine (J.T.S, V.Y.-Y.), Department of Pharmacology (C.E.C, I.V), and Biophysics Graduate Group (J.M., A.M.E, K.R.D), University of California Davis, Davis, California;

Department of Pharmacology (K.F.), Tokushima Bunri University, Tokushima, Japan

[1]I.V. and V.Y.-Y. are co-senior authors

Supplemental Material Methods

S1 Text

Rosetta movemap files and generalized command lines

Radial movemap

RESIDUE   1 148 NO

RESIDUE 173 177 NO

RESIDUE 192 202 NO

RESIDUE 253 412 NO

RESIDUE 437 441 NO

RESIDUE 456 466 NO

RESIDUE 517 676 NO

RESIDUE 701 705 NO

RESIDUE 720 730 NO

RESIDUE 781 940 NO

RESIDUE 965 969 NO

RESIDUE 984 994 NO

RESIDUE 1045 1056 NO

RESIDUE 149 172 BBCHI

RESIDUE 178 191 BBCHI

RESIDUE 203 252 BBCHI

RESIDUE 413 436 BBCHI

RESIDUE 442 455 BBCHI

RESIDUE 467 516 BBCHI

RESIDUE 677 700 BBCHI

RESIDUE 706 719 BBCHI

RESIDUE 731 780 BBCHI

RESIDUE 941 964 BBCHI

RESIDUE 970 983 BBCHI

RESIDUE 995 1044 BBCHI

Regional movemap

RESIDUE 141 264 BBCHI

RESIDUE 405 528 BBCHI

RESIDUE 669 792 BBCHI

RESIDUE 993 1056 BBCHI

RESIDUE 1 140 NO

RESIDUE 265 404 NO

RESIDUE 529 668 NO

RESIDUE 793 992 NO

Command line flags for Rosetta Relax

```
-in:file:movemap movemap.map \
-score:weights   membrane_highres_Menv_smooth_cart.wts   \
-relax:constrain_relax_to_start_coords   \
-relax:jump_move true \
-relax:dualspace true \
-relax:min_type  lbfgs_armijo_nonmonotone   \
```

-symmetry:symmetry_definition file.symm \

-symmetry:initialize_rigid_body_dofs \

-default_max_cycles 200 \

-membrane \

-membrane:no_interpolate_Mpair \

-membrane:Menv_penalties \

-use_input_sc \

-ignore_unrecognized_res \

-ex1 \

-ex2 \

-ex2aro \

-extrachi_cutoff=3 \

-nstruct 100 \

-out:prefix mrelax-symm- \

-out:file:silent output.silent \

-out:file:silent_struct_type binary \

-mute all

Rosetta Ligand docking

Command line flags

-in:path:database $ROSETTA/rosetta3.11/main/database \

-in:file:s drug-pdb-file.pdb \

-parser:protocol RosettaScripts.xml \

-beta \

-nstruct 2 \

-extra_res_fa params-file.params \

-use_input_sc \

-ignore_waters false \

-ex1 \

-ex2 \

-ex2aro \

-extrachi_cutoff 3 \

```
-chemical:exclude_patches LowerDNA UpperDNA Cterm_amidation SpecialRotamer VirtualBB ShoveBB
   VirtualDNAPhosphate VirtualNTerm CTermConnect sc_orbitals pro_hydroxylated_case1
   pro_hydroxylated_case2 ser_phosphorylated thr_phosphorylated tyr_phosphorylated tyr_sulfated
   lys_dimethylated lys_monomethylated lys_trimethylated lys_acetylated glu_carboxylated cys_acetylated
   tyr_diiodinated N_acetylated C_methylamidated MethylatedProteinCterm \
-out:prefix docking_ligand \
-out:file:silent output.silent \
-out:file:silent_struct_type binary \
-mute all \
```

S1 XML Script
RosettaScripts for GALigand Dock

```
<ROSETTASCRIPTS>
    <SCOREFXNS>
     <ScoreFunction name="genpot_cart" weights="beta_genpot_cart">
       <Reweight scoretype="coordinate_constraint" weight="0.1"/>
     </ScoreFunction>
     <ScoreFunction name="genpot" weights="beta_genpot"/>
     <ScoreFunction  name="genpot_cart_soft"  weights="beta_genpot_cart">
       <Reweight scoretype="fa_rep" weight="0.2"/>
     </ScoreFunction>
    </SCOREFXNS>
    <FILTERS>
     <LigInterfaceEnergy name="LigInterface" scorefxn="genpot_cart" confidence="0.0" />
     <DSasa name="DSASA" lower_threshold="0.0" upper_threshold="1.0" confidence="0.0" />
    </FILTERS>
    <MOVERS>

     <GALigandDock name="dock" runmode="VSH" scorefxn="genpot_cart_soft" grid_step="0.25" padding="5.0" hashsize="8.0"
subhash="3" final_exact_minimize="bbsc1" random_oversample="10" rotprob="0.9" rotEcut="100" sidechains="auto"
initial_pool="input.pdb">
       <Stage repeats="10" npool="50" pmut="0.2" smoothing="0.375" rmsdthreshold="2.5" maxiter="50" pack_cycles="100"
ramp_schedule="0.1,1.0"/>
       <Stage repeats="10" npool="50" pmut="0.2" smoothing="0.375" rmsdthreshold="1.5" maxiter="50" pack_cycles="100"
ramp_schedule="0.1,1.0"/>
     </GALigandDock>
     <MultiplePoseMover name="add_filter">
       <ROSETTASCRIPTS>
         <SCOREFXNS>
```

```
        <ScoreFunction name="genpot_cart" weights="beta_genpot_cart">
          <Reweight scoretype="coordinate_constraint" weight="0.1"/>
        </ScoreFunction>
        <ScoreFunction name="genpot" weights="beta_genpot"/>
        <ScoreFunction name="genpot_cart_soft" weights="beta_genpot_cart">
          <Reweight scoretype="fa_rep" weight="0.2"/>
        </ScoreFunction>
        </SCOREFXNS>
        <FILTERS>
          <LigInterfaceEnergy name="LigInterface" scorefxn="genpot_cart"
           confidence="0.0"/>
          <DSasa name="DSASA" lower_threshold="0.0" upper_threshold="1.0"
           confidence="0.0"/>
        </FILTERS>
        <PROTOCOLS>
                  <Add filter_name="LigInterface" />
        </PROTOCOLS>
      </ROSETTASCRIPTS>
    </MultiplePoseMover>
  </MOVERS>
  <PROTOCOLS>
    <Add mover_name="dock" />
    <Add mover_name="add_filter" />
  </PROTOCOLS>
  <OUTPUT />
</ROSETTASCRIPTS>
```

Supplemental Fig. 1: Comparison of input and top 20 relaxed wild type (WT) models. **A**) Overlay of model 1 (m1, orange stick) and model 2 (m2, blue stick) WT input models showing a top-view of F627 and surrounding residues (top), orientation of R582 on the S5P turret helix (top, inset), side-view of the SF backbone (middle), and backside-view of the hydrophobic fenestration region under the SF along S5 and S6 segments (bottom). **B**) Top 20 relaxed WT m1 radial (left panel) and regional (right panel) models showing conformational variation in F627 and surrounding residues (top) and fenestration region (bottom). Input structures are in orange stick, with all other residues colored according to specific domains as in **Figure 1**. **C**) Same as **B** but for WT m2 models.

Supplemental Fig. 2: Comparison of top 20 relaxed S641A mutant models. **A**) Overlay of S641A m1 radial (left panels) and regional (right panels) models showing the SF (top) and fenestration (bottom) regions. Residue colors as in **Supplemental Fig. 1**. **B**) Same as **A** but for S641A m2 models. **C**) SF region of WT m1 radial models with a constrained S620-N629 hydrogen bond. **D**) SF region of S641A m1 regional models with a constrained S620-N629 hydrogen bond.

Supplemental Fig. 3: Comparison of SF region only for top 20 relaxed S641T m1 (**A**) and m2 (**B**) models for both radial (top) and regional (bottom) models. Residue colors as in **Supplemental Fig. 1**.

Supplemental Fig. 4: Comparison of top 20 relaxed S620T mutant models. **A**) Overlay of S620T m1 radial (left panels) and regional (right panels) models showing the SF (top) and fenestration (bottom) regions. Residue colors as in **Supplemental Fig. 1**. **B**) Same as **A** but for S620T m2 models.

Supplemental Fig. 1

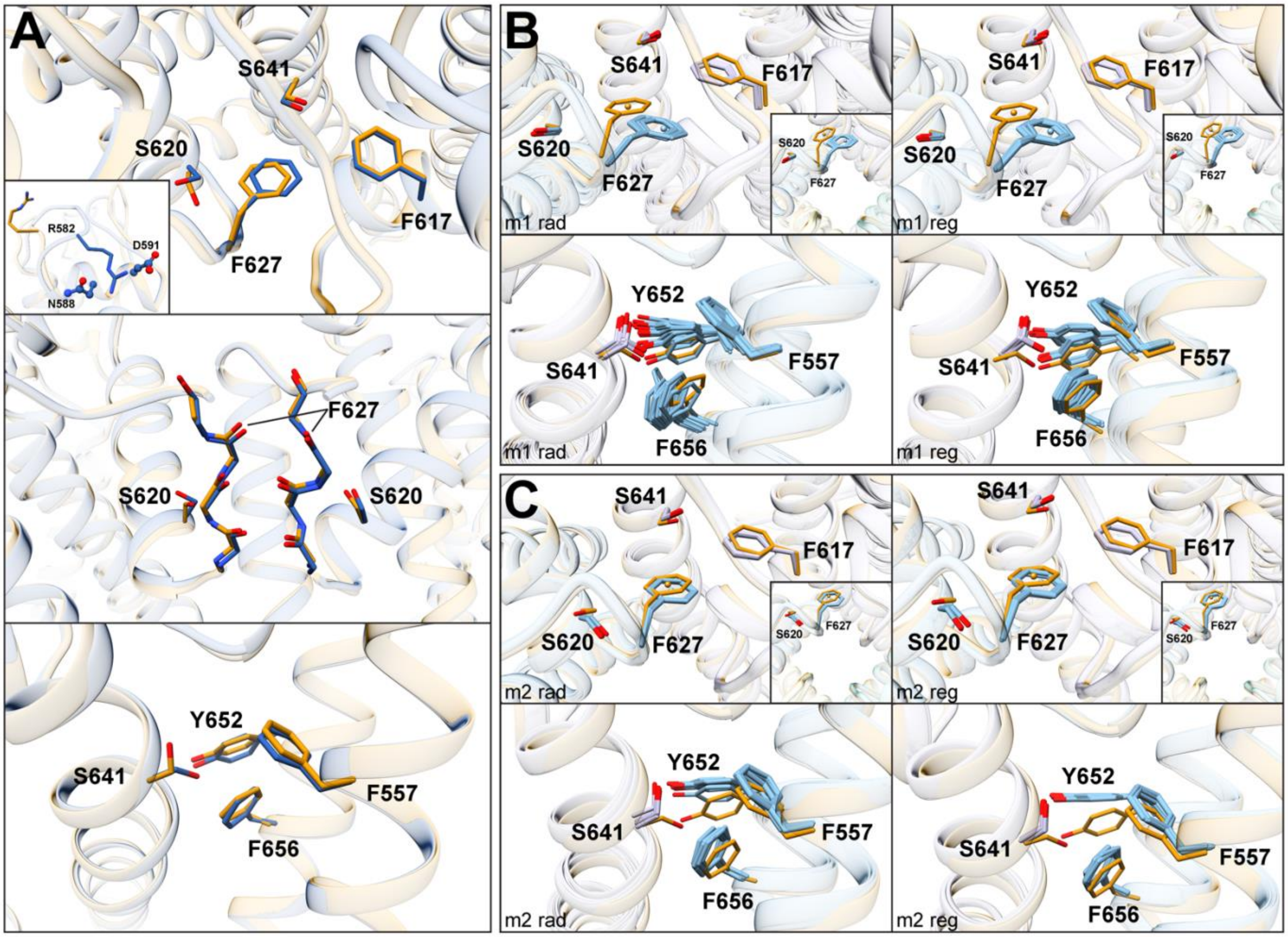

Supplemental Fig. 2

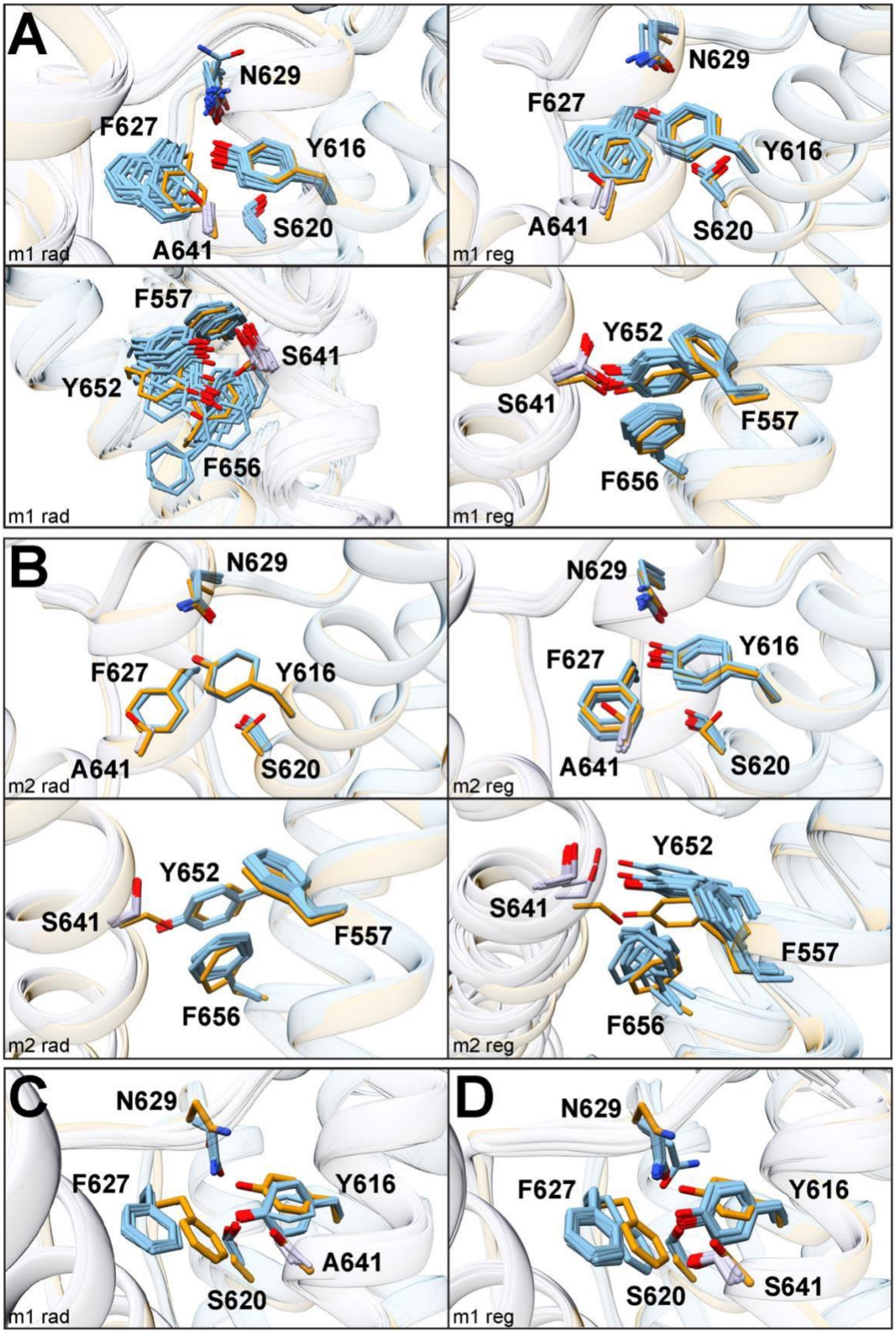

Supplemental Fig. 3

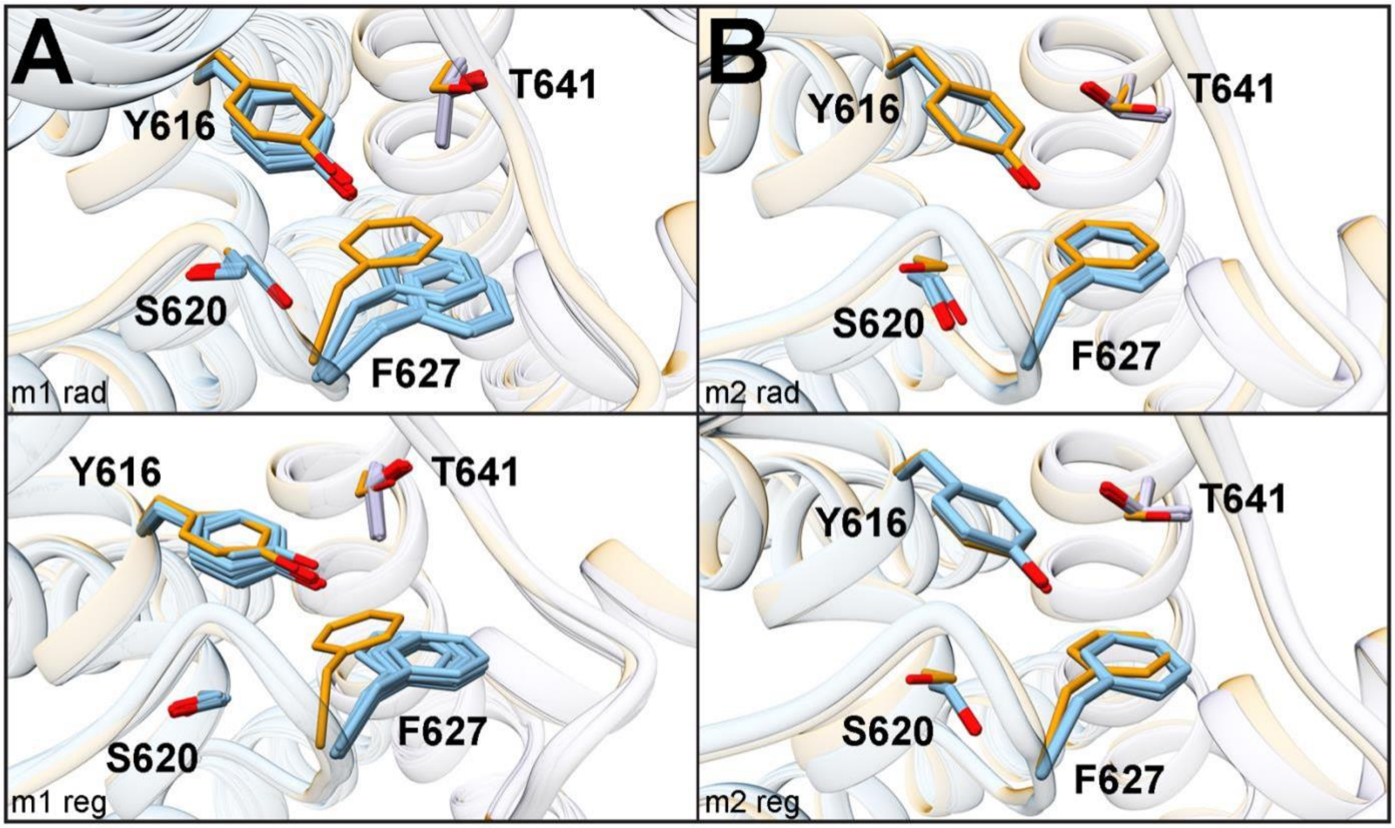

Supplemental Fig. 4

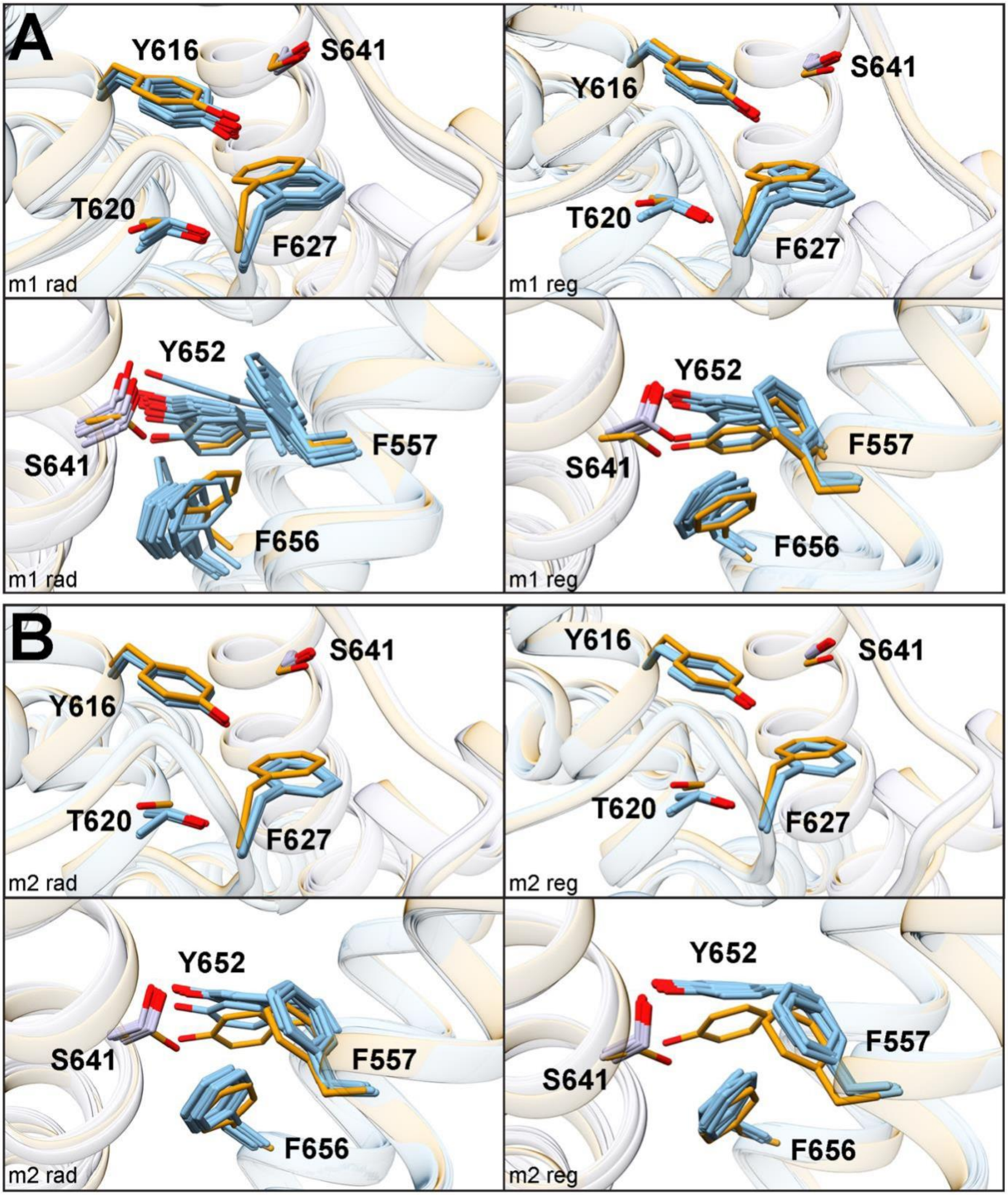

Supplemental Table 1

	dft-(0)	dft-(1)	ter-(0)	terf-(1)	E4031-(0)	E4031-(1)
WT/m1	-56.875	-56.099	-56.645	-56.568	-56.224	-56.78
WT/m2	-56.422	-56.426	-56.537	-58.08	-56.432	-56.477
S641A/m1	-52.13	-52.032	-52.185	-52.511	-54.18	-53.23
S641A/m2	-59.779	-59.573	-59.677	-60.119	-59.801	-59.759
S641T/m1	-58.595	-59.045	-58.464	-58.309	-56.65	-59.794
S641T/m2	-54.008	-53.966	-54.145	-54.673	-53.886	-54.34
S620T/m1	-58.648	-58.34	-58.209	-58.359	-58.301	-59.439
S620T/m2	-57.889	-57.774	-58.238	-58.357	-57.953	-58.75

-50
-51
-52
-53
-54
-55
-56
-57
-58
-59
-60

Summary of the minimum Rosetta energy scores (in Rosetta energy units) for the hERG – ligand models shown in Figures 2, 4, and 6. Values in the table are color-coded according to the color scale shown on the right side.
